# Scarcity of research on psychological or psychiatric states using validated questionnaires in low- and middle-income countries: A ChatGPT-assisted bibliometric analysis and national case study on some psychometric properties

**DOI:** 10.7189/jogh.13.04102

**Published:** 2023-10-02

**Authors:** Duško Rudan, Darko Marčinko, Dunja Degmečić, Nenad Jakšić

**Affiliations:** 1 Department of Psychiatry and Psychological Medicine, University Hospital Centre Zagreb, Zagreb, Croatia; 2 Faculty of Medicine, University of Zagreb, Department of Psychiatry and Psychological Medicine, University Hospital Centre Zagreb, Zagreb, Croatia; 3 Faculty of Medicine, J. J. Strossmayer University of Osijek, Department of Psychiatry, University Hospital Centre Osijek, Osijek, Croatia; 4 Department of Psychiatry and Psychological Medicine, University Hospital Centre Zagreb, Zagreb, Croatia

## Abstract

**Background:**

It is vital to assess whether research on psychological or psychiatric states using validated questionnaires is still lagging in low- and middle-income countries and to what degree, and to continue to assess the psychometric properties of the most informative questionnaires.

**Methods:**

We performed a bibliometric analysis of Web of Science Core Collection for all years to determine the number of studies performed in each country that used an inventory or a questionnaire on aggression, anxiety, depression, borderline personality, narcissism, self-harm, shame, or childhood trauma. We conducted a simple observational analysis of distributions by countries to derive the main overall conclusions, assisted by ChatGPT to test its ability to summarise and interpret this type of information. We also carried out a study in Croatia to examine some psychometric properties of five commonly used questionnaires, using Cronbach’s α coefficient and zero-order correlations.

**Results:**

We observed a concentration of research activity in a few high-income countries, primarily the United States and several European nations, suggesting a robust research infrastructure and a strong emphasis on studying psychological and psychiatric states within their population. In contrast, low- and middle-income countries were notably under-represented in research on psychological and psychiatric states, although the gap seems to be closing in some countries. Turkey, Iran, Brazil, South Africa, Mexico, India, Malaysia and Pakistan have been consistently contributing an increasing number of studies and catching up with the most research-intensive high-income countries. The national case study in Croatia confirmed adequate psychometric properties of the most frequently used questionnaires.

**Conclusions:**

Addressing research gaps in low- and middle-income countries is crucial, because relying solely on research from high-income countries may not fully capture the nuances of psychological and psychiatric states within diverse populations. To bridge this gap, it is essential to prioritise mental health research in low-resource settings, provide training and resources to local researchers, and establish international collaborations. Such efforts can lead to the development of culturally valid questionnaires, an improved understanding of psychological and psychiatric states in diverse contexts, and the creation of effective interventions to promote mental well-being on a global scale.

The study of psychological and psychiatric states is crucial for understanding and addressing the mental health challenges faced by individuals worldwide. Recent systematic reviews have shown that mental health disorders are still one of the leading causes of global disability, with depressive and anxiety disorders causing 49.4 and 44.5 million disability-adjusted life-years (DALYs) worldwide, respectively [1,2]. However, despite the growing recognition of mental health as a global concern, there remains a significant scarcity of research focused on low- and middle-income countries (LMICs). These regions are home to a substantial proportion of the world's population, and the burden of mental health disorders is particularly pronounced [1,3]. Inadequate attention to mental health research in LMICs perpetuates disparities in access to evidence-based interventions and contributes to a lack of culturally sensitive approaches for mental health promotion and treatment [4].

Here we present a bibliometric analysis of the scarcity of research on psychological and psychiatric states using validated inventories or questionnaires in LMICs. We provide an overview of the research output in this field, enabling us to examine the geographical distribution of studies and national-level overall productivity across eight inventories and questionnaires, aiming to highlight the urgent need for increased attention and investment in mental health research in LMICs [5].

We also present a national case study in Croatia that further examines the psychometric properties of these inventories and questionnaires. In the case of mental health research in Croatia, previous empirical studies utilising such self-report instruments have assessed levels and correlates of depression and anxiety [6,7], pathological narcissism [8,9], shame [8], borderline personality [10], childhood trauma [11], and aggression [12]. For example, certain cross-cultural differences were obtained between post-war transitional Croatian society and some Western countries in regard to depression [13] and pathological narcissism [14]. This part of our study aims to provide a contextualised understanding of the challenges and opportunities that influence the validity of research on psychological and psychiatric states, taking into account cultural factors.

## METHODS

### Global bibliometric analysis of the differences between high-income countries and low- and middle-income countries

We searched Web of Science Core Collection from inception to 6 July 2023 (day of search), using the following search terms: aggression, anxiety, depression, borderline personality, narcissism, self-harm, shame, and childhood trauma. We paired each term with “AND (inventory or questionnaire)” to return all studies, distributed by country. We selected these terms because they relate to very common types of mental health disorders and personality dysfunctions and because we could provide national data in the context of broader literature. The classification of countries into high-income and low- and middle-income was based on most recent World Bank data [15]. The search results showing the top 20 high-income countries and top 20 low- and middle-income countries are presented in **Table 1**, **Table 2**, **Table 3**, and **Table 4**. Each table was then entered in the ChatGPT with a prompt to interpret and discuss the results.

**Table 1:** Availability of studies on aggression (left) and anxiety (right) in high-income vs low- and middle-income countries (according to Web of Science Core Collection, all years, search conducted on July 6, 2023)*

Aggression and (inventory or questionnaire) (n = 9551)		Anxiety and (inventory or questionnaire) (n = 93 983)	
**High income**	**Low- and middle-income**	**High income**	**Low- and middle-income**
USA: 3049 (31.92)	People’s Republic of China: 566 (5.93)	USA: 22 125 (23.54)	People’s Republic of China: 6792 (7.23)
UK: 1006 (10.53)	Turkey: 308 (3.22)	UK: 10 723 (11.41)	Turkey: 4497 (4.78)
Spain: 702 (7.35)	Iran: 195 (2.04)	Australia: 6091 (6.48)	Iran: 3203 (3.41)
Canada: 667 (6.98)	Brazil: 141 (1.48)	Germany: 6052 (6.44)	Brazil: 2399 (2.55)
Netherlands: 524 (5.49)	Russia: 141 (1.48)	Canada: 5474 (5.82)	India: 1509 (1.61)
Germany: 518 (5.42)	India: 77 (0.81)	Netherlands: 4986 (5.31)	Malaysia: 735 (0.78)
Australia: 459 (4.81)	Mexico: 74 (0.77)	Spain: 4458 (4.74)	South Africa: 639 (0.68)
Italy: 421 (4.41)	South Africa: 64 (0.67)	Italy: 4131 (4.40)	Pakistan: 608 (0.65)
Sweden: 226 (2.37)	Colombia: 53 (0.55)	Sweden: 2820 (3.00)	Russia: 580 (0.62)
France: 185 (1.94)	Pakistan: 47 (0.49)	France: 2686 (2.86)	Mexico: 574 (0.61)
Poland: 175 (1.83)	Malaysia: 40 (0.42)	Japan: 2331 (2.48)	Egypt: 425 (0.45)
Japan: 162 (1.70)	Argentina: 37 (0.39)	South Korea: 1942 (2.07)	Indonesia: 358 (0.38)
Switzerland: 161 (1.69)	Serbia: 36 (0.38)	Norway: 1858 (1.98)	Serbia: 324 (0.34)
Norway: 160 (1.68)	Ukraine: 32 (0.34)	Switzerland: 1618 (1.72)	Thailand: 321 (0.34)
Israel: 156 (1.63)	Nigeria: 31 (0.32)	Israel: 1427 (1.52)	Nigeria: 311 (0.33)
South Korea: 145 (1.52)	Egypt: 21 (0.22)	Poland: 1423 (1.51)	Argentina: 263 (0.28)
Belgium: 133 (1.39)	Indonesia: 21 (0.22)	Belgium: 1271 (1.35)	Colombia: 257 (0.27)
Finland: 130 (1.36)	Thailand: 20 (0.21)	Denmark: 1247 (1.33)	Jordan: 256 (0.27)
Portugal: 120 (1.26)	Jordan: 18 (0.19)	Taiwan: 1165 (1.24)	Ethiopia: 248 (0.26)
Austria: 98 (1.03)	Bosnia & Herzegovina: 15 (0.16)	Finland: 989 (1.05)	Bangladesh: 195 (0.21)

*Data presented as n (%) unless otherwise specified.

**Table 2:** Availability of studies on depression (left) and borderline personality (right) in high-income vs low- and middle-income countries (according to Web of Science Core Collection, all years, search conducted on July 6, 2023)*

Depression and (inventory or questionnaire) (n = 121 136)		Borderline personality and (inventory or questionnaire) (n = 3185)	
**High income**	**Low- and middle-income**	**High income**	**Low- and middle-income**
USA: 35 019 (28.91)	People’s Republic of China: 8358 (6.90)	USA: 1128 (35.42)	Turkey 89 (2.79)
UK: 11 980 (9.89)	Turkey: 4816 (3.98)	Germany: 437 (13.72)	People’s Republic of China: 85 (2.67)
Germany: 8195 (6.77)	Iran: 3334 (2.75)	UK: 305 (9.58)	Iran: 65 (2.04)
Australia: 7534 (6.22)	Brazil: 3274 (2.70)	Canada: 237 (7.44)	Brazil: 30 (0.94)
Canada: 7150 (5.90)	India: 1807 (1.49)	Italy: 209 (6.56)	Mexico: 18 (0.57)
Netherlands: 5875 (4.85)	South Africa: 947 (0.78)	Netherlands: 187 (5.87)	South Africa:13 (0.41)
Italy: 4881 (4.03)	Malaysia: 822 (0.68)	Australia: 173 (5.43)	Egypt: 11 (0.35)
Spain: 4686 (3.87)	Mexico: 746 (0.62)	Spain: 161 (5.05)	India: 11 (0.35)
Japan: 3539 (2.92)	Pakistan: 740 (0.61)	Switzerland: 128 (4.02)	Russia: 10 (0.31)
Sweden: 3471 (2.87)	Egypt: 545 (0.45)	France: 111 (3.49)	Argentina: 7 (0.22)
France: 3212 (2.65)	Russia: 526 (0.43)	Belgium: 87 (2.73)	Thailand: 7 (0.22)
South Korea: 3193 (2.64)	Thailand: 509 (0.42)	Norway: 70 (2.20)	Pakistan: 6 (0.19)
Norway: 2344 (1.94)	Nigeria: 487 (0.40)	Austria: 60 (1.88)	Serbia: 5 (0.16)
Switzerland: 2262 (1.87)	Ethiopia: 453 (0.37)	Sweden: 59 (1.85)	Bosnia & Herzegovina: 3 (0.09)
Poland: 1877 (1.55)	Serbia: 406 (0.34)	Denmark: 56 (1.76)	Morocco: 3 (0.09)
Finland: 1866 (1.54)	Argentina: 327 (0.27)	Poland: 39 (1.22)	Colombia: 2 (0.06)
Denmark: 1810 (1.49)	Colombia: 327 (0.27)	Hungary: 34 (1.07)	Ethiopia: 2 (0.06)
Taiwan: 1704 (1.41)	Indonesia: 316 (0.26)	Israel: 31 (0.97)	Barbados: 1 (0.03)
Belgium: 1637 (1.35)	Jordan: 285 (0.24)	Japan: 30 (0.94)	Ecuador: 1 (0.03)
Israel: 1593 (1.32)	Bangladesh: 245 (0.20)	Finland: 27 (0.85)	Iraq: 1 (0.03)

*Data presented as country: n (%).

**Table 3:** Availability of studies on narcissism (left) and self-harm (right) in high-income vs low- and middle-income countries (according to Web of Science Core Collection, all years, search conducted on July 6, 2023)*

Narcissism and (inventory or questionnaire) (n = 1939)		Self-harm and (inventory or questionnaire) (n = 1815)	
**High income**	**Low- and middle-income**	**High income**	**Low- and middle-income**
USA: 828 (42.70)	People’s Republic of China: 95 (4.90)	USA: 496 (27.33)	People’s Republic of China: 118 (6.50)
Germany: 187 (9.64)	Turkey: 43 (2.22)	UK: 419 (23.09)	Turkey 47 (2.59)
UK: 187 (9.64)	Iran: 35 (1.81)	Australia: 200 (11.02)	Iran: 29 (1.60)
Canada: 158 (8.15)	Russia: 24 (1.24)	Canada: 116 (6.39)	India: 28 (1.54)
Australia: 99 (5.11)	Serbia: 19 (0.98)	Germany: 99 (5.45)	Russia: 19 (1.05)
Italy: 83 (4.28)	Malaysia: 16 (0.83)	Italy 77 (4.24)	Brazil: 17 (0.94)
Poland: 81 (4.18)	Brazil: 13 (0.67)	Sweden: 69 (3.80)	Pakistan: 16 (0.88)
Netherlands: 70 (3.61)	Pakistan: 11 (0.57)	Netherlands: 66 (3.64)	Malaysia: 14 (0.77)
Israel: 56 (2.89)	India: 10 (0.52)	Ireland: 62 (3.42)	Mexico: 14 (0.77)
Spain: 53 (2.73)	Argentina: 9 (0.46)	Norway: 59 (3.25)	South Africa: 11 (0.61)
Austria: 36 (1.86)	Indonesia: 6 (0.31)	Spain: 59 (3.25)	Nigeria: 10 (0.55)
Switzerland: 30 (1.55)	Mexico: 5 (0.26)	Belgium: 48 (2.64)	Sri Lanka: 9 (0.50)
Japan: 24 (1.24)	Ukraine: 5 (0.26)	France: 43 (2.37)	Bulgaria: 5 (0.28)
Sweden: 22 (1.13)	Jordan: 4 (0.21)	Japan: 42 (2.31)	Ethiopia: 5 (0.28)
Croatia: 20 (1.03)	Thailand: 4 (0.21)	Finland: 34 (1.87)	Indonesia: 5 (0.28)
Norway: 19 (0.98)	Egypt: 3 (0.15)	New Zealand: 30 (1.65)	Jordan: 5 (0.28)
Belgium: 18 (0.93)	Lebanon: 3 (0.15)	Israel: 29 (1.60)	Tunisia: 5 (0.28)
Denmark: 18 (0.93)	South Africa: 3 (0.15)	Switzerland: 29 (1.60)	Bangladesh: 4 (0.22)
South Korea: 18 (0.93)	Bosnia & Herzegovina: 2 (0.10)	Taiwan: 29 (1.60)	Colombia: 4 (0.22)
Czech Republic: 13 (0.67)	Georgia: 2 (0.10)	Denmark: 28 (1.54)	Morocco: 4 (0.22)

*Data presented as country: n (%).

**Table 4:** Availability of studies on shame (left) and childhood trauma (right) in high-income vs low- and middle-income countries (according to Web of Science Core Collection, all years, search conducted on July 6, 2023)*

Shame and (inventory or questionnaire) (n = 1658)		Childhood trauma and (inventory or questionnaire) (n = 511)	
**High income**	**Low- and middle-income**	**High income**	**Low- and middle-income**
USA: 484 (29.19)	People’s Republic of China: 70 (4.22)	USA: 1722 (33.65)	People’s Republic of China: 457 (8.93)
UK: 193 (11.64)	Iran: 45 (2.71)	Germany: 613 (11.98)	Turkey: 320 (6.25)
Canada: 126 (7.60)	Turkey: 40 (2.41)	UK: 492 (9.61)	Brazil: 179 (3.50)
Germany: 126 (7.60)	Brazil: 36 (2.17)	Canada: 386 (7.54)	South Africa: 84 (1.64)
Australia: 117 (7.06)	India: 15 (0.90)	Netherlands: 327 (6.39)	Iran: 60 (1.17)
Netherlands: 89 (5.37)	Mexico: 13 (0.78)	Australia: 245 (4.79)	India: 29 (0.57)
Italy: 66 (3.98)	South Africa: 12 (0.72)	Italy: 193 (3.77)	Mexico: 18 (0.35)
Portugal: 62 (3.74)	Nigeria: 10 (0.60)	Switzerland: 164 (3.20)	Serbia: 13 (0.25)
Spain: 49 (2.96)	Russia: 10 (0.60)	France: 130 (2.54)	Thailand: 11 (0.21)
Sweden: 48 (2.90)	Pakistan: 9 (0.54)	South Korea: 125 (2.44)	Egypt: 10 (0.20)
Israel: 47 (2.83)	Jordan: 7 (0.42)	Spain: 125 (2.44)	Malaysia: 10 (0.20)
Norway: 44 (2.65)	Indonesia: 6 (0.36)	Norway: 121 (2.36)	Nigeria: 10 (0.20)
France: 39 (2.35)	Malaysia: 6 (0.36)	Sweden: 114 (2.23)	Argentina: 9 (0.18)
Poland: 36 (2.17)	Thailand: 6 (0.36)	Israel: 104 (2.03)	Bosnia & Herzegovina: 9 (0.18)
Switzerland: 34 (2.05)	Philippines: 5 (0.30)	Austria: 83 (1.62)	Kenya: 9 (0.18)
Belgium: 31 (1.87)	Bangladesh: 4 (0.24)	Belgium: 69 (1.35)	Jordan: 8 (0.16)
Japan: 26 (1.57)	Argentina: 3 (0.18)	Poland: 64 (1.25)	Lebanon: 8 (0.16)
South Korea: 20 (1.21)	Colombia: 3 (0.18)	Denmark: 63 (1.23)	Russia: 8 (0.16)
Austria: 18 (1.09)	Egypt: 3 (0.18)	Japan: 62 (1.21)	Tanzania: 8 (0.16)
Denmark: 18 (1.09)	Ethiopia: 3 (0.18)	Ireland: 59 (1.15)	Uganda: 8 (0.16)

*Data presented as country: n (%).

### National case study in Croatia

Croatia’s income classification by the World Bank’s data [15] has changed over time. Before 2008, Croatia was classified as an upper-middle-income country. From 2008 to the present, which was also reclassified in 2019, it has been considered a high-income country.

The research was conducted on 111 participants aged between 19 and 62 years, diagnosed with borderline personality disorder (BPD) (ICD-10 code = F60.3 [16]). Twenty-eight (25.2%) of the participants were male, with an average age of 41.2 years (standard deviation (SD) = 10.3), while the remaining 83 (74.8%) were female, with an average age of 36.5 years (SD = 12.9). We categorised age into three groups based on the first and third quartiles of the age distribution within the sample: the youngest group consisted of the first 25% (18-26 years), the adult group included the next 50% (27-49 years), and the oldest group comprised the remaining 25% (50-62 years). The average age was 23.1 (n = 30 (SD = 2.76)) years for the youngest participants, 38.1 (N = 57, SD = 7.2) years for adults, and 55.1 (N = 24, SD = 3.2) years for the oldest group.

In this sample, we tested the reliability of five commonly used questionnaires or inventories (The Childhood Trauma Questionnaire-Short Form (CTQ-SF) [17,18], Pathological Narcissism Inventory (PNI) [19], Experience of Shame Scale (ESS) [20], The Aggression Questionnaire (AQ) [21], and Borderline Personality Questionnaire (BPQ) [22]) using Cronbach’s alpha, which is a statistical measure used to assess the internal consistency or reliability of a scale or a set of items in a questionnaire or test. It ranges between 0 and 1, with a higher value indicating greater internal consistency, meaning that the items in the scale are more closely related to each other and are measuring the same concept. Cronbach's alpha was calculated using the standard procedure, taking a score from each scale item and correlating it with the total score for each observation, after which the resulting correlations are compared with the variance for all individual item scores [23].

## RESULTS

### Bibliometric analysis of the global use of the inventory or questionnaire on aggression, anxiety, depression, borderline personality, narcissism, self-harm, shame, and childhood trauma

The results of the analysis showed a clear disparity in the number of research studies utilising validated questionnaires to detect various psychological or psychiatric states in different countries. Depression and anxiety were by far the most researched states, with 121 136 and 93 983 studies published across all years, respectively (**Table 1**, **Table 2**, **Table 3**, and **Table 4**). Interestingly, for depression, although the USA was clearly ahead of the rest of the world with 35 019 studies, which is nearly a third of the total global output, the number of studies in some of the LMIC was comparable to the most productive high-income countries (HICs): China (1^st^ among LMICs) was more productive than Germany (3^rd^ among HICs), Turkey (2^nd^ among LMICs) than Spain (8^th^ among HICs), and Iran and Brazil (3^rd^ and 4^th^ among LMICs) than France (11^th^ in HICs). These results are somewhat surprising. Even Jordan and Bangladesh (19^th^ and 20^th^ in LMICs) accounted for 285 and 245 studies, respectively. This shows that inventories and questionnaires for depression have spread all over the world and there is sufficient information to assess it, even in most middle-income countries. Only low-income countries in Africa and South-East Asia showed a real scarcity of output.

We observed a similar situation with anxiety inventories and questionnaires. Again, the USA was first in overall productivity, as its researchers published nearly a quarter of the global output. However, China was again ahead of the 3^rd^ ranked country among HICs (Australia), while Turkey was ahead of 7^th^-ranked HIC (Spain), and Iran and Brazil were comparable to Sweden, France, and Japan. We again found a surprisingly large number of studies in large low-income countries, such as Pakistan (n = 608), Ethiopia (n = 248), and Bangladesh (n = 195). This suggests that depression and anxiety are very well-researched in many places, and although the number of studies per capita is certainly much greater in HICs, as is the number of citations per paper compared LMICs (data not shown), the difference in overall intensity in research activity is not striking – it is clear that research on depression and anxiety in populations living in LMIC is growing.

However, the situation seemed to be much different regarding the six remaining inventories or questionnaires. In all years, there were 9551 studies on aggression, 3185 on borderline personality, 1939 on narcissism, 1815 on self-harm, 1658 on shame, and 5118 on childhood trauma. The early adoption of these questionnaires was much greater in HICs than in LMICs, with a real scarcity among the latter. China, Turkey, Iran, Brazil, Russia, and India were the only LMIC countries that consistently had a number of studies comparable to second-tier productivity among HICs, while other LMICs countries were still lagging behind and were only beginning to adopt these questionnaires. Therefore, there is real scarcity of information on aggression, borderline personality, narcissism, self-harm, shame, and childhood trauma for most of the world’s population, particularly in low-resource settings.

There are some specific findings that deserve mention. The United States emerged as the country with the highest number of publications in this area, and was typically followed by the UK. Spain, Germany, Canada, Australia, Netherlands, and Italy are also consistently highly productive countries in this area of research, while France and especially Japan and South Korea were less productive than could be expected by their population sizes and level of development of their research infrastructure. However, Poland had surprisingly high productivity among high-income countries, as the only consistently prominent example from Eastern Europe.

Turkey was a positive example of productivity among LMICs; it was the 2^nd^ most productive LMIC behind China for six out of eight inventories and questionnaires, and was ranked 1^st^ (ahead of China) for the number of studies on borderline personality. Iran (ranked 2^nd^ once and 3^rd^ six more times among LMICs), as well as Ethiopia and Bangladesh (consistently among the top 20 LMICs) were also found to be good examples of high productivity and output.

We found wide variation in the number of research studies utilising validated questionnaires to detect anxiety in different countries. The United States emerged as the country with the highest number of publications.

### The use of ChatGPT 3.5 to interpret the results

We presented **Table 1**, **Table 2**, **Table 3**, and **Table 4**, to ChatGPT 3.5, accompanied with explanations provided in table legends, and asked it to summarise and interpret the results (**Box 1**). We wanted to explore ChatGPT’s ability to analyse and interpret this kind of data, based on a recent demonstration on its capacity to assist in writing a research article [24]. We also tested it table-by-table and questionnaire-by-questionnaire (data not shown). ChatGPT wrote many very general sentences typical of scientific papers’ results and discussion sections, but it proved rather unreliable. It had difficulties with a correct understanding of the meaning of two separate columns. It did not always classify individual countries in HIC vs. LMIC group correctly, making misclassification errors in both directions. Also, it focused mainly on the HIC situation and was unable to adequately describe the LMIC context. This reflects the shortcomings of its current version in being used to assist in writing scientific papers. Furthermore, the references it provided mainly did not exist, which is another interesting characteristic of its output. Consequently, we recommend caution in using ChatGPT to summarise and interpret large amounts of data in a research paper, especially if this requires interpreting complex tables, a need for a link to a third source (e.g. the World Bank’s categorisation of countries into HIC vs LMIC), or appropriate and correct referencing. These are areas where we expect ChatGPT to improve in its future iterations.

Box 1 **Output from the ChatGPT 3.5**

Prompt: “Here are four tables that show the results of bibliometric analysis of the use of the inventory or questionnaire on aggression, anxiety, depression, borderline personality, narcissism, self-harm, shame and childhood trauma. For each of the eight inventories and questionnaires, the tables show the top 20 high-income countries and the top 20 low- and middle-income countries, ranked by the number of published papers. Please analyse and discuss the results.”

ChatGPT 3.5: “The provided tables present the results of bibliometric analysis on the use of various inventories and questionnaires related to aggression, anxiety, depression, borderline personality, narcissism, self-harm, shame, and childhood trauma. The tables are divided into two sections: one for high-income countries and the other for low- and middle-income countries. The results are based on the number of published papers related to each inventory or questionnaire.

Let's analyze and discuss the key findings from each table:

Table 1: Availability of studies on aggression and anxiety

The USA and China are the top two countries with the highest number of published papers on both aggression and anxiety.

High-income countries dominate the list of top contributors for both aggression and anxiety, with only a few low- and middle-income countries making it to the top 20.

Table 2: Availability of studies on depression and borderline personality

The USA and China lead in the number of published papers related to depression, while Turkey and Iran follow closely.

Similar to the aggression and anxiety inventories, high-income countries are more represented in the top 20 contributors for depression and borderline personality, though Turkey and Brazil from low- and middle-income countries also appear.

Table 3: Availability of studies on narcissism and self-harm

The USA and Germany are the top contributors to research on narcissism, while the USA and China lead in the self-harm category.

There is a relatively more balanced representation of low- and middle-income countries in the top 20 contributors for narcissism and self-harm, with countries like Turkey, Iran, and Malaysia making appearances.

Table 4: Availability of studies on shame and childhood trauma

The USA is the leading contributor to research on both shame and childhood trauma, followed by China for shame and Turkey for childhood trauma.

The representation of low- and middle-income countries is slightly better in the top 20 contributors for shame and childhood trauma, with countries like Turkey, Brazil, South Africa, and India making appearances.

Overall, the analysis reveals a significant concentration of research in high-income countries across all inventories and questionnaires. These countries, particularly the USA and China, dominate the research landscape in almost every category. However, there is evidence of some contribution from low- and middle-income countries as well, particularly in areas like self-harm, narcissism, and childhood trauma.

The findings suggest that there is a need to encourage and support more research on mental health and related issues in low- and middle-income countries to gain a comprehensive understanding of these phenomena across diverse populations. Additionally, collaborative efforts between high-income and low- and middle-income countries could enhance research outcomes and promote global mental health initiatives.”

### National case study on the psychometric properties in Croatia

We evaluated five of these inventories and questionnaires in Croatia, to explore if translations are still culturally appropriate and measure the studied traits reasonably well, thus making them applicable in a broad global context. Internal consistency of the CTQ-SF [17,18], with its 25 items, measured by a Cronbach's α coefficient of 0.908, means it could be considered a reliable instrument for assessing overall childhood trauma (**Table 5**). The Cronbach's α coefficients for the subscales were determined based on their components. All subscales exhibited high reliability, except for physical neglect, which fell slightly below the threshold of reliability. Notably, the CTQ-SF subscales do not follow a normal distribution. Items CTQ10, CTQ16, and CTQ22 were excluded in the process. The subscales of the CTQ-SF can be considered reliable in Croatian context (**Table 5**).

**Table 5:** Subscales of the Childhood Trauma Questionnaire – Short Form

Sub-scale			
**Code**	**Name**	**Sum of components**	**Cronbach’s α**
CTQ-1	Emotional Abuse	CTQ3 + CTQ8 + CTQ14 + CTQ18 + CTQ25	0.862
CTQ-2	Physical Abuse	CTQ9 + CTQ11 + CTQ12 + CTQ15 + CTQ17	0.812
CTQ-3	Sexual Abuse	CTQ20 + CTQ21 + CTQ23 + CTQ24 + CTQ27	0.920
CTQ-4	Emotional Neglect	(6-CTQ5) + (6-CTQ7) + (6-CTQ13) + (6-CTQ19) + (6-CTQ28)	0.876
CTQ-5	Physical Neglect	CTQ1 + (6-CTQ2) + CTQ4 + CTQ6 + (6-CTQ26)	0.651
CTQ-6	Minimisation/Denial	Sum with one point for each answer „5“ on CTQ10, CTQ16 and CTQ22	0.855

CTQ – Childhood Trauma Questionnaire

The PNI self-assessment questionnaire for pathological narcissism [18,19] consists of 52 items, with responses coded from 0 to 5. The high Cronbach's α coefficient value of 0.963 indicates the questionnaire's high reliability in assessing self-assessment of pathological narcissism. To assess the components of the self-assessment questionnaire for pathological narcissism, 10 subscales were formed (**Table 6**). The reliability of these aggregated items was slightly reduced, but still remained above the very high threshold of 0.9, as measured by Cronbach's α coefficient. Omitting any of the subscales did not affect the reliability of the self-assessment questionnaire for pathological narcissism.

**Table 6:** Subscales of the Pathological Narcissism Inventory

Sub-scale			
**Code**	**Name**	**Sum of components**	**Cronbach’s α**
PNI-1	Contingent Self-Esteem	(PNI2 + PNI5 + PNI8 + PNI16 + PNI19 + PNI30 + PNI32 + PNI36 + PNI40 + PNI41 + PNI47 + PNI48)/12	0.918
PNI-2	Exploitativeness	(PNI4 + PNI10 + PNI15 + PNI23 + PNI35)/5	0.934
PNI-3	Self-Sacrificing Self-Enhancement	(PNI6 + PNI22 + PNI25 + PNI33 + PNI39 + PNI43)/6	0.925
PNI-4	Hiding the Self	(PNI7 + PNI9 + PNI13 + PNI28 + PNI44 + PNI46 + PNI50)/7	0.925
PNI-5	Grandiose Fantasy	(PNI1 + PNI14 + PNI26 + PNI31 + PNI42 + PNI45 + PNI49)/7	0.917
PNI-6	Devaluing	(PNI3 + PNI17 + PNI21 + PNI24 + PNI27 + PNI34 + PNI51)/7	0.940
PNI-7	Entitlement Rage	(PNI11 + PNI12 + PNI18 + PNI20 + PNI29 + PNI37 + PNI38 + PNI52)/8	0.917
PNI-8	Grandiosity	(PNI-5 × 7 + PNI-2 × 5 + PNI-3 × 6)/18	0.915
PNI-9	Vulnerability	(PNI-1 × 12 + PNI-4 × 7 + PNI-6 × 7 + PNI-7 × 8)/34	0.911
PNI-10	Pathological Narcissism	(PNI-8 × 18 + PNI-9 × 34)/52	0.911

PNI – Pathological Narcissism Inventory

In assessing the ESS [20], the high Cronbach's α coefficient value of 0.957 indicates its high reliability (**Table 7**). Three subscales were formed to assess the components of the self-assessment questionnaire for shame experience. The reliability of these aggregated items was slightly reduced, but remained above the very high threshold of 0.920, as measured by Cronbach's α coefficient (**Table 7**). Omitting any of the items in any of the cases did not alter the reliability of the shame experience scale.

**Table 7:** Subscales of the Experience of Shame Scale

Sub-scale			
**Code**	**Name**	**Sum of components**	**Cronbach’s α**
ESS-1	Characterological Shame	ESS1 + ESS2 + … + ESS12	0.922
ESS-2	Behavioural Shame	ESS13 + ESS14 + … + ESS21	0.922
ESS-3	Bodily Shame	ESS22 + ESS23 + ESS24 + ESS25	0.924
ESS-Total	Total Experience of Shame	ESS1 + ESS2 + … + ESS25	0.957

ESS – Experience of Shame Scale

The subscale values for the AQ [21] had a Cronbach's α coefficient of 0.922, indicating the scale’s high reliability in assessing aggression. To assess the components of the aggression questionnaire and their reliability, four subscales were formed (**Table 8**). The reliability of these aggregated items has been significantly reduced, especially in the case of verbal aggression (AQ-VA), as its reliability was only 0.658. The reliability of the other subscales had also been reduced, but they remained at an acceptable level.

**Table 8:** Subscales of the Aggression Questionnaire

Sub-scale			
**Code**	**Name**	**Sum of components**	**Cronbach’s α**
AQ-PA	Physical Aggression	AQ3 + AQ5 + AQ6 + AQ9 + AQ11 + AQ14 + (6-AQ17) + AQ22 + AQ23	0.858
AQ-VA	Verbal Aggression	AQ10 + AQ19 + AQ21 + AQ27 + AQ29	0.658
AQ-A	Anger	(6-AQ1) + AQ7 + AQ16 + AQ20 + AQ24 + AQ26 + AQ28	0.825
AQ-H	Hostility	AQ2 + AQ4 + AQ8 + AQ12 + AQ13 + AQ15 + AQ18 + AQ25	0.814
AQ-Total	Total Aggression	AQ-PA + AQ-VA + AQ-A + AQ-H	0.909

AQ – Aggression Questionnaire

According to the BPQ [22], the assessment procedure involves summing selected items into nine subscales (**Table 9**). The items that define each subscale are provided as well. Some of the items are included in the sum in an inverted form, marked additionally with “-I”. The reliability of these subscales, measured by Cronbach's α coefficients, ranged from 0.708 to 0.869, indicating a satisfactory level of reliability, from sufficiently to highly reliable. The total score (BPQ-Total) is the sum of all nine subscales. Its reliability, determined by Cronbach's α coefficient of all 80 items, was slightly higher than the reliability of the components, with a value of 0.901 (**Table 9**). Omitting individual items only slightly affected the reliability.

**Table 9:** Subscales of the Borderline Personality Questionnaire

Sub-scale			
**Code**	**Name**	**Sum of components**	**Cronbach’s α**
BPQ-1	Impulsivity	BPQ1 + BPQ10-I + BPQ26 + BPQ34 + BPQ42 + BPQ57 + BPQ64 + BPQ68 + BPQ71	0.708
BPQ-2	Affective Instability	BPQ2 + BPQ11 + BPQ19 + BPQ27 + BPQ35 + BPQ43-I + BPQ49 + BPQ58 + BPQ65 + BPQ72	0.831
BPQ-3	Abandonment	BPQ3 + BPQ12 + BPQ20 + BPQ28-I + BPQ44 + BPQ50 + BPQ59 + BPQ66 + BPQ73 + BPQ78	0.791
BPQ-4	Relationships	BPQ4-I + BPQ13 + BPQ21 + BPQ29 + BPQ36 + BPQ45-I + BPQ51 + BPQ60-I	0.887
BPQ-5	Self-Image	BPQ5 + BPQ14 + BPQ37 + BPQ46 + BPQ52-I + BPQ61 + BPQ67-I + BPQ70 + BPQ74	0.832
BPQ-6	Suicide/Self-Mutilation	BPQ6 + BPQ15 + BPQ22 + BPQ30 + BPQ38 + BPQ53-I + BPQ75	0.874
BPQ-7	Emptiness	BPQ7 + BPQ16 + BPQ23 + BPQ31 + BPQ39 + BPQ54-I + BPQ62 + BPQ69 + BPQ76 + BPQ79	0.822
BPQ-8	Intense Anger	BPQ8-I + BPQ17 + BPQ24 + BPQ32-I + BPQ40 + BPQ47 + BPQ55 + BPQ63 + BPQ77 + BPQ80	0.869
BPQ-9	Quasi-Psyhotic States	BPQ9 + BPQ18 + BPQ25 + BPQ33 + BPQ41 + BPQ48-I + BPQ56	0.747
BPQ-Total	Borderline Personality Questionnaire Total	BPQ-1 + BPQ-2 + BPQ-3 + BPQ-4 + BPQ-5 + BPQ-6 + BPQ-7 + BPQ-8 + BPQ-9	0.901

BPQ – Borderline Personality Questionnaire

We also carried out zero-order correlations between the investigated psychological/psychiatric variables, with an emphasis on the associations of CTQ-SF scales with the rest of the used questionnaires (i.e. PNI, ESS, AQ, BPQ) (**Online Supplementary Document**).

## DISCUSSION

Despite the growing interest in bibliometric analyses of the progress in the fields of psychology and psychiatry, there is still a lack of topical and focused analyses [25-32], particularly those assessing the situation in less developed populations. To our knowledge, this is one of the first studies to compare research intensity in this field between HICs and LMICs.

The distribution of research studies on all the validated inventories and questionnaires, apart from the two most commonly used – i.e. on anxiety and depression, demonstrates a concentration of research activity in high-income countries, primarily the USA and several European nations (**Table 1**, **Table 2**, **Table 3**, and **Table 4**). The predominance of the USA in terms of publication output suggests a robust research infrastructure and a strong emphasis on studying psychological and psychiatric traits within their population. This may be attributed to many underlying factors, such as the availability of research funding, established research institutions, and a culture of prioritising mental health research. The high number of research studies in the UK, Germany, Spain, Australia, Canada, and the People's Republic of China also indicates significant attention given to studying those traits using validated questionnaires. All of those countries possess supportive research environments, well-established academic networks, and research initiatives that prioritize mental health-related issues. Their sizable research output may also be influenced by their population sizes.

Notably, several low- and middle-income countries, such as Turkey, Spain, Brazil, and China, also contributed a substantial number of research studies. This indicates a growing recognition of the importance of mental health research and the need to understand and address these challenges in LMICs. The distribution of these studies suggests efforts to bridge the research gap between HICs and LMICs in studying psychological and psychiatric traits.

However, most LMICs are notably underrepresented in research on the six validated questionnaires that do not include anxiety and depression. The limited number of studies from these countries raises concerns about the lack of research capacity, funding constraints, and potential cultural or language barriers that hinder the adoption of standardised questionnaires in measuring psychological or psychiatric traits. This scarcity of research in low- and middle-income countries emphasises the urgent need for greater investment in mental health research and the development of culturally sensitive tools and frameworks to understand aggression in diverse populations.

Addressing the research gaps in low- and middle-income countries is crucial for several reasons. First, the prevalence and manifestation of psychological and psychiatric issues may differ across cultures and socioeconomic contexts. Therefore, relying solely on research conducted in high-income countries may not fully capture the nuances of psychological and psychiatric issues within diverse populations. Second, a lack of research in these countries hampers the development and implementation of evidence-based interventions and policies tailored to their specific needs. Finally, by increasing research capacity in low- and middle-income countries, it becomes possible to foster international collaborations and exchange knowledge and best practices in addressing aggression globally.

To bridge the research gap, it is essential to prioritise mental health research in underrepresented regions, provide training and resources to local researchers, and establish collaborations between high-income and low- and middle-income countries. It is also crucial to acknowledge that the number of research studies alone does not determine the quality or impact of the research conducted. However, the variation in the number of studies across countries provides valuable insights into the research landscape and highlights the areas where more attention and resources are required. Overall, this analysis underscores the need for a more balanced and inclusive approach to mental health research.

## CONCLUSIONS

The findings from this analysis emphasise the need for a comprehensive and global approach to researching narcissism, borderline personality disorder, childhood trauma, aggression, self-harm and shame. By expanding research efforts to a wider range of countries, it becomes possible to gain a more nuanced understanding of these complex traits and develop culturally sensitive interventions to address and mitigate its negative impacts.

To the best of our knowledge, we conducted the first analysis of ChatGPT’s potential to summarise the results of bibliometric evaluation of the inventories and questionnaires in psychology and psychiatry. We showed that it is not quite ready yet for the full-scale implementation with this particular aim, but it is possible that it might become more useful in time, with further iterations.

Finally, the addition of a national-level case study in Croatia, where five of the eight inventories and questionnaires were evaluated, showed that they were highly reliable when translated and applied in a different cultural context. Therefore, alongside the instruments detecting anxiety and depression, which have already achieved widespread global use, there is a future for increased and expanded use of the six instruments narcissism, borderline personality disorder, childhood trauma, aggression, self-harm and shame in LMICs, and we would like to encourage the roll-out of this research in the coming years.

## Additional material


Online Supplementary Document
