# Supplementary material for: Scarcity of research on psychological or psychiatric states using validated questionnaires in low- and middle-income countries: A ChatGPT-assisted bibliometric analysis and national case study on some psychometric properties
Source: J Glob Health. 2023 Oct 2;13:04102. doi: 10.7189/jogh.13.04102 (PMC10543016; doi:10.7189/jogh.13.04102)
Supplement: Online Supplementary Document [file jogh-13-04102-s001.pdf]

SUPPLEMENTARY ONLINE MATERIAL

Correlations between variables

Since the basis for testing the hypotheses is formed by correlations between the variables, an intercorrelation matrix of Pearson correlation coefficients for 41 potential aggregated variables is provided for testing possible mediation effects in line with specific hypotheses that constitute the backbone of this research. In the five parts of the correlation matrix, only the Pearson correlation coefficients are displayed. Correlations that are statistically significant are marked in red. All "red" coefficients are statistically significant with a margin of error of 5% or less, between pairs of variables from the corresponding row or column, identified by the respective variable codes in which the coefficient is found. As evident from the tables shown below, the variables of the aggregated subscales and total scales of the utilized questionnaires are almost invariably positively correlated with each other. The exception is the socio-economic status of the participants (SES), which significantly correlates only with CTQ-2, AQ-A, AQ-H, AQ-Total, SHI-Total, BPQ-1, and BAI-Total variables. This fact limits the possibility of testing SES as a mediator only in relation to SHI-Total and BPQ-Total. For this reason, possible correlations of the most important components of socio-economic status (socio-demographic and clinical) with all variables were tested. It is evident that among all components, only SES-A (length of hospital treatment - hospitalization) has a significant number of correlations with other variables. Therefore, it is the only socio-economic status component that could potentially act as a mediator in the relationships between variable pairs in the models for investigating the research objectives. **Tables S1-S6** show the matrices of correlations.

Table S1. Correlation matrices between variables – Part 1.

Variable	SES	CTQ-1	CTQ-2	CTQ-3	CTQ-4	CTQ-5	CTQ-6	CTQ-Total
SES	1,000	0,045	0,286	0,182	0,050	0,183	0,013	0,178
CTQ-1	0,045	1,000	0,767	0,378	0,698	0,489	-0,369	0,891
CTQ-2	0,286	0,767	1,000	0,450	0,511	0,543	-0,216	0,852
CTQ-3	0,182	0,378	0,450	1,000	0,255	0,258	-0,062	0,575
CTQ-4	0,050	0,698	0,511	0,255	1,000	0,609	-0,421	0,815
CTQ-5	0,183	0,489	0,543	0,258	0,609	1,000	-0,295	0,718
CTQ-6	0,013	-0,369	-0,216	-0,062	-0,421	-0,295	1,000	-0,364
CTQ-Total	0,178	0,891	0,852	0,575	0,815	0,718	-0,364	1,000
PNI-1	0,107	0,420	0,280	0,137	0,277	0,171	-0,249	0,347
PNI-2	0,097	0,057	0,037	0,053	-0,103	0,009	0,092	0,010
PNI-3	0,030	0,342	0,210	0,078	0,176	0,149	-0,090	0,258
PNI-4	0,095	0,388	0,258	0,057	0,357	0,227	-0,107	0,349
PNI-5	0,160	0,320	0,236	0,110	0,182	0,079	-0,119	0,253
PNI-6	0,052	0,236	0,065	-0,004	0,282	0,065	-0,067	0,185
PNI-7	0,166	0,308	0,247	0,070	0,168	0,081	-0,094	0,241
PNI-8	0,130	0,326	0,222	0,107	0,134	0,104	-0,070	0,242
PNI-9	0,129	0,418	0,259	0,085	0,329	0,160	-0,171	0,343
PNI-10	0,136	0,409	0,260	0,097	0,280	0,149	-0,146	0,327
ESS-1	0,067	0,413	0,303	0,115	0,339	0,299	-0,233	0,389
ESS-2	0,022	0,314	0,240	0,081	0,275	0,196	-0,199	0,296
ESS-3	0,059	0,381	0,300	0,249	0,369	0,332	-0,209	0,422
ESS-Total	0,055	0,413	0,311	0,145	0,359	0,300	-0,240	0,403
AQ-PA	0,099	0,375	0,464	0,109	0,278	0,344	-0,073	0,410
AQ-VA	0,149	0,202	0,282	-0,042	0,151	0,184	0,082	0,208
AQ-A	0,205	0,311	0,323	-0,052	0,324	0,258	-0,110	0,315
AQ-H	0,194	0,438	0,397	0,083	0,415	0,270	-0,190	0,432
AQ-Total	0,193	0,420	0,458	0,045	0,371	0,332	-0,109	0,433
SHI-Total	0,191	0,508	0,446	0,320	0,377	0,396	-0,181	0,530
BPQ-1	0,194	0,302	0,281	0,159	0,341	0,396	-0,179	0,377
BPQ-2	0,059	0,271	0,256	0,147	0,241	0,181	-0,164	0,288
BPQ-3	0,046	0,527	0,423	0,234	0,398	0,295	-0,263	0,498
BPQ-4	0,084	0,373	0,375	0,140	0,271	0,223	-0,199	0,367
BPQ-5	-0,041	0,311	0,235	0,192	0,299	0,193	-0,181	0,324
BPQ-6	0,028	0,461	0,390	0,233	0,365	0,386	-0,179	0,476
BPQ-7	0,023	0,296	0,279	0,121	0,294	0,193	-0,157	0,314
BPQ-8	0,078	0,306	0,310	0,092	0,272	0,272	-0,171	0,328
BPQ-9	0,135	0,126	0,131	0,173	0,153	0,242	-0,134	0,201
BPQ-Total	0,085	0,458	0,414	0,223	0,402	0,355	-0,248	0,485
BAI-Total	0,198	0,298	0,412	0,242	0,199	0,352	-0,255	0,378
BDI2-Total	0,153	0,262	0,276	0,013	0,266	0,276	-0,147	0,287

Table S2. Correlation matrices between variables – Part 2.

Variable	PNI-1	PNI-2	PNI-3	PNI-4	PNI-5	PNI-6	PNI-7	PNI-8
SES	0,107	0,097	0,030	0,095	0,160	0,052	0,166	0,130
CTQ-1	0,420	0,057	0,342	0,388	0,320	0,236	0,308	0,326
CTQ-2	0,280	0,037	0,210	0,258	0,236	0,065	0,247	0,222
CTQ-3	0,137	0,053	0,078	0,057	0,110	-0,004	0,070	0,107
CTQ-4	0,277	-0,103	0,176	0,357	0,182	0,282	0,168	0,134
CTQ-5	0,171	0,009	0,149	0,227	0,079	0,065	0,081	0,104
CTQ-6	-0,249	0,092	-0,090	-0,107	-0,119	-0,067	-0,094	-0,070
CTQ-Total	0,347	0,010	0,258	0,349	0,253	0,185	0,241	0,242
PNI-1	1,000	0,321	0,565	0,493	0,723	0,511	0,705	0,718
PNI-2	0,321	1,000	0,306	0,259	0,445	0,125	0,505	0,685
PNI-3	0,565	0,306	1,000	0,514	0,501	0,345	0,564	0,764
PNI-4	0,493	0,259	0,514	1,000	0,549	0,472	0,494	0,583
PNI-5	0,723	0,445	0,501	0,549	1,000	0,432	0,718	0,882
PNI-6	0,511	0,125	0,345	0,472	0,432	1,000	0,415	0,410
PNI-7	0,705	0,505	0,564	0,494	0,718	0,415	1,000	0,774
PNI-8	0,718	0,685	0,764	0,583	0,882	0,410	0,774	1,000
PNI-9	0,881	0,373	0,614	0,706	0,760	0,767	0,820	0,774
PNI-10	0,873	0,499	0,698	0,702	0,842	0,687	0,848	0,892
ESS-1	0,629	0,065	0,225	0,437	0,463	0,540	0,389	0,360
ESS-2	0,678	-0,002	0,336	0,516	0,480	0,495	0,414	0,391
ESS-3	0,464	0,032	0,136	0,375	0,362	0,387	0,165	0,260
ESS-Total	0,682	0,039	0,274	0,503	0,499	0,549	0,392	0,390
AQ-PA	0,214	0,216	0,014	0,281	0,236	0,022	0,391	0,203
AQ-VA	0,293	0,390	0,173	0,252	0,392	0,228	0,584	0,406
AQ-A	0,384	0,220	0,179	0,357	0,412	0,357	0,590	0,364
AQ-H	0,618	0,198	0,284	0,510	0,512	0,486	0,575	0,453
AQ-Total	0,460	0,287	0,188	0,432	0,459	0,323	0,629	0,416
SHI-Total	0,485	0,171	0,111	0,337	0,306	0,267	0,319	0,264
BPQ-1	0,361	0,251	0,126	0,241	0,378	0,220	0,317	0,335
BPQ-2	0,551	0,101	0,271	0,366	0,444	0,504	0,487	0,380
BPQ-3	0,544	0,106	0,253	0,367	0,300	0,423	0,470	0,296
BPQ-4	0,433	0,105	0,154	0,293	0,367	0,347	0,456	0,293
BPQ-5	0,561	-0,072	0,126	0,336	0,220	0,397	0,168	0,145
BPQ-6	0,439	0,096	0,097	0,287	0,285	0,227	0,290	0,223
BPQ-7	0,578	-0,057	0,191	0,359	0,308	0,412	0,298	0,223
BPQ-8	0,513	0,236	0,246	0,319	0,466	0,389	0,591	0,425
BPQ-9	0,313	0,261	0,133	0,257	0,321	0,304	0,238	0,311
BPQ-Total	0,665	0,145	0,248	0,435	0,472	0,499	0,515	0,400
BAI-Total	0,306	0,006	0,236	0,381	0,263	0,281	0,238	0,237
BDI2-Total	0,494	0,046	0,218	0,324	0,310	0,342	0,269	0,269

Table S3. Correlation matrices between variables – Part 3.

Variable	PNI-9	PNI-10	ESS-1	ESS-2	ESS-3	ESS-Total	AQ-PA	AQ-VA
SES	0,129	0,136	0,067	0,022	0,059	0,055	0,099	0,149
CTQ-1	0,418	0,409	0,413	0,314	0,381	0,413	0,375	0,202
CTQ-2	0,259	0,260	0,303	0,240	0,300	0,311	0,464	0,282
CTQ-3	0,085	0,097	0,115	0,081	0,249	0,145	0,109	-0,042
CTQ-4	0,329	0,280	0,339	0,275	0,369	0,359	0,278	0,151
CTQ-5	0,160	0,149	0,299	0,196	0,332	0,300	0,344	0,184
CTQ-6	-0,171	-0,146	-0,233	-0,199	-0,209	-0,240	-0,073	0,082
CTQ-Total	0,343	0,327	0,389	0,296	0,422	0,403	0,410	0,208
PNI-1	0,881	0,873	0,629	0,678	0,464	0,682	0,214	0,293
PNI-2	0,373	0,499	0,065	-0,002	0,032	0,039	0,216	0,390
PNI-3	0,614	0,698	0,225	0,336	0,136	0,274	0,014	0,173
PNI-4	0,706	0,702	0,437	0,516	0,375	0,503	0,281	0,252
PNI-5	0,760	0,842	0,463	0,480	0,362	0,499	0,236	0,392
PNI-6	0,767	0,687	0,540	0,495	0,387	0,549	0,022	0,228
PNI-7	0,820	0,848	0,389	0,414	0,165	0,392	0,391	0,584
PNI-8	0,774	0,892	0,360	0,391	0,260	0,390	0,203	0,406
PNI-9	1,000	0,977	0,639	0,666	0,442	0,677	0,265	0,420
PNI-10	0,977	1,000	0,579	0,608	0,403	0,616	0,258	0,438
ESS-1	0,639	0,579	1,000	0,835	0,549	0,946	0,253	0,151
ESS-2	0,666	0,608	0,835	1,000	0,589	0,938	0,183	0,033
ESS-3	0,442	0,403	0,549	0,589	1,000	0,729	0,132	-0,039
ESS-Total	0,677	0,616	0,946	0,938	0,729	1,000	0,227	0,078
AQ-PA	0,265	0,258	0,253	0,183	0,132	0,227	1,000	0,613
AQ-VA	0,420	0,438	0,151	0,033	-0,039	0,078	0,613	1,000
AQ-A	0,523	0,497	0,391	0,265	0,113	0,323	0,618	0,744
AQ-H	0,686	0,644	0,584	0,601	0,404	0,616	0,490	0,464
AQ-Total	0,568	0,547	0,438	0,358	0,213	0,405	0,840	0,799
SHI-Total	0,447	0,409	0,613	0,516	0,600	0,641	0,375	0,204
BPQ-1	0,362	0,372	0,507	0,388	0,385	0,490	0,341	0,288
BPQ-2	0,611	0,565	0,501	0,499	0,379	0,529	0,286	0,340
BPQ-3	0,575	0,511	0,571	0,540	0,389	0,582	0,394	0,319
BPQ-4	0,486	0,446	0,392	0,423	0,269	0,421	0,412	0,360
BPQ-5	0,477	0,389	0,607	0,626	0,529	0,665	0,168	0,047
BPQ-6	0,396	0,359	0,642	0,558	0,572	0,665	0,399	0,242
BPQ-7	0,532	0,455	0,605	0,610	0,458	0,642	0,270	0,152
BPQ-8	0,578	0,557	0,489	0,390	0,267	0,455	0,550	0,599
BPQ-9	0,351	0,356	0,241	0,234	0,311	0,281	0,270	0,205
BPQ-Total	0,677	0,619	0,702	0,660	0,540	0,728	0,476	0,396
BAI-Total	0,366	0,341	0,344	0,367	0,336	0,390	0,352	0,230
BDI2-Total	0,459	0,419	0,575	0,556	0,330	0,577	0,345	0,230

Table S4. Correlation matrices between variables – Part 4.

Variable	AQ-A	AQ-H	AQ-Total	SHI-Total	BPQ-1	BPQ-2	BPQ-3	BPQ-4
SES	0,205	0,194	0,193	0,191	0,194	0,059	0,046	0,084
CTQ-1	0,311	0,438	0,420	0,508	0,302	0,271	0,527	0,373
CTQ-2	0,323	0,397	0,458	0,446	0,281	0,256	0,423	0,375
CTQ-3	-0,052	0,083	0,045	0,320	0,159	0,147	0,234	0,140
CTQ-4	0,324	0,415	0,371	0,377	0,341	0,241	0,398	0,271
CTQ-5	0,258	0,270	0,332	0,396	0,396	0,181	0,295	0,223
CTQ-6	-0,110	-0,190	-0,109	-0,181	-0,179	-0,164	-0,263	-0,199
CTQ-Total	0,315	0,432	0,433	0,530	0,377	0,288	0,498	0,367
PNI-1	0,384	0,618	0,460	0,485	0,361	0,551	0,544	0,433
PNI-2	0,220	0,198	0,287	0,171	0,251	0,101	0,106	0,105
PNI-3	0,179	0,284	0,188	0,111	0,126	0,271	0,253	0,154
PNI-4	0,357	0,510	0,432	0,337	0,241	0,366	0,367	0,293
PNI-5	0,412	0,512	0,459	0,306	0,378	0,444	0,300	0,367
PNI-6	0,357	0,486	0,323	0,267	0,220	0,504	0,423	0,347
PNI-7	0,590	0,575	0,629	0,319	0,317	0,487	0,470	0,456
PNI-8	0,364	0,453	0,416	0,264	0,335	0,380	0,296	0,293
PNI-9	0,523	0,686	0,568	0,447	0,362	0,611	0,575	0,486
PNI-10	0,497	0,644	0,547	0,409	0,372	0,565	0,511	0,446
ESS-1	0,391	0,584	0,438	0,613	0,507	0,501	0,571	0,392
ESS-2	0,265	0,601	0,358	0,516	0,388	0,499	0,540	0,423
ESS-3	0,113	0,404	0,213	0,600	0,385	0,379	0,389	0,269
ESS-Total	0,323	0,616	0,405	0,641	0,490	0,529	0,582	0,421
AQ-PA	0,618	0,490	0,840	0,375	0,341	0,286	0,394	0,412
AQ-VA	0,744	0,464	0,799	0,204	0,288	0,340	0,319	0,360
AQ-A	1,000	0,623	0,884	0,340	0,403	0,532	0,485	0,450
AQ-H	0,623	1,000	0,796	0,474	0,397	0,513	0,635	0,656
AQ-Total	0,884	0,796	1,000	0,441	0,437	0,505	0,568	0,579
SHI-Total	0,340	0,474	0,441	1,000	0,571	0,438	0,624	0,395
BPQ-1	0,403	0,397	0,437	0,571	1,000	0,406	0,392	0,375
BPQ-2	0,532	0,513	0,505	0,438	0,406	1,000	0,570	0,565
BPQ-3	0,485	0,635	0,568	0,624	0,392	0,570	1,000	0,734
BPQ-4	0,450	0,656	0,579	0,395	0,375	0,565	0,734	1,000
BPQ-5	0,243	0,458	0,300	0,551	0,305	0,490	0,552	0,306
BPQ-6	0,402	0,540	0,499	0,834	0,513	0,425	0,639	0,443
BPQ-7	0,346	0,539	0,416	0,575	0,384	0,648	0,630	0,437
BPQ-8	0,795	0,567	0,750	0,440	0,467	0,690	0,564	0,503
BPQ-9	0,225	0,378	0,335	0,317	0,323	0,225	0,297	0,266
BPQ-Total	0,606	0,723	0,680	0,722	0,618	0,786	0,836	0,720
BAI-Total	0,340	0,451	0,429	0,384	0,229	0,455	0,434	0,395
BDI2-Total	0,421	0,520	0,475	0,538	0,364	0,487	0,594	0,388

Table S5. Correlation matrices between variables – Part 5.

Variable	BPQ-5	BPQ-6	BPQ-7	BPQ-8	BPQ-9	BPQ-Total	BAI-Total	BDI2-Total
SES	-0,041	0,028	0,023	0,078	0,135	0,085	0,198	0,153
CTQ-1	0,311	0,461	0,296	0,306	0,126	0,458	0,298	0,262
CTQ-2	0,235	0,390	0,279	0,310	0,131	0,414	0,412	0,276
CTQ-3	0,192	0,233	0,121	0,092	0,173	0,223	0,242	0,013
CTQ-4	0,299	0,365	0,294	0,272	0,153	0,402	0,199	0,266
CTQ-5	0,193	0,386	0,193	0,272	0,242	0,355	0,352	0,276
CTQ-6	-0,181	-0,179	-0,157	-0,171	-0,134	-0,248	-0,255	-0,147
CTQ-Total	0,324	0,476	0,314	0,328	0,201	0,485	0,378	0,287
PNI-1	0,561	0,439	0,578	0,513	0,313	0,665	0,306	0,494
PNI-2	-0,072	0,096	-0,057	0,236	0,261	0,145	0,006	0,046
PNI-3	0,126	0,097	0,191	0,246	0,133	0,248	0,236	0,218
PNI-4	0,336	0,287	0,359	0,319	0,257	0,435	0,381	0,324
PNI-5	0,220	0,285	0,308	0,466	0,321	0,472	0,263	0,310
PNI-6	0,397	0,227	0,412	0,389	0,304	0,499	0,281	0,342
PNI-7	0,168	0,290	0,298	0,591	0,238	0,515	0,238	0,269
PNI-8	0,145	0,223	0,223	0,425	0,311	0,400	0,237	0,269
PNI-9	0,477	0,396	0,532	0,578	0,351	0,677	0,366	0,459
PNI-10	0,389	0,359	0,455	0,557	0,356	0,619	0,341	0,419
ESS-1	0,607	0,642	0,605	0,489	0,241	0,702	0,344	0,575
ESS-2	0,626	0,558	0,610	0,390	0,234	0,660	0,367	0,556
ESS-3	0,529	0,572	0,458	0,267	0,311	0,540	0,336	0,330
ESS-Total	0,665	0,665	0,642	0,455	0,281	0,728	0,390	0,577
AQ-PA	0,168	0,399	0,270	0,550	0,270	0,476	0,352	0,345
AQ-VA	0,047	0,242	0,152	0,599	0,205	0,396	0,230	0,230
AQ-A	0,243	0,402	0,346	0,795	0,225	0,606	0,340	0,421
AQ-H	0,458	0,540	0,539	0,567	0,378	0,723	0,451	0,520
AQ-Total	0,300	0,499	0,416	0,750	0,335	0,680	0,429	0,475
SHI-Total	0,551	0,834	0,575	0,440	0,317	0,722	0,384	0,538
BPQ-1	0,305	0,513	0,384	0,467	0,323	0,618	0,229	0,364
BPQ-2	0,490	0,425	0,648	0,690	0,225	0,786	0,455	0,487
BPQ-3	0,552	0,639	0,630	0,564	0,297	0,836	0,434	0,594
BPQ-4	0,306	0,443	0,437	0,503	0,266	0,720	0,395	0,388
BPQ-5	1,000	0,559	0,788	0,394	0,260	0,725	0,449	0,649
BPQ-6	0,559	1,000	0,571	0,502	0,304	0,757	0,364	0,568
BPQ-7	0,788	0,571	1,000	0,464	0,289	0,813	0,464	0,657
BPQ-8	0,394	0,502	0,464	1,000	0,273	0,761	0,406	0,520
BPQ-9	0,260	0,304	0,289	0,273	1,000	0,463	0,398	0,381
BPQ-Total	0,725	0,757	0,813	0,761	0,463	1,000	0,553	0,711
BAI-Total	0,449	0,364	0,464	0,406	0,398	0,553	1,000	0,592
BDI2-Total	0,649	0,568	0,657	0,520	0,381	0,711	0,592	1,000

Table S6. Spearman's correlations of the chosen components of the SES questionnaire.

Variable	SES1	SES2	SES3A	SES4	SES7	SES-A	SES-B
CTQ-1	-0,056	0,099	-0,108	0,075	-0,036	0,098	0,182
CTQ-2	-0,108	0,117	-0,004	-0,083	0,021	0,177	0,221
CTQ-3	0,032	0,070	-0,092	0,037	-0,110	0,070	0,056
CTQ-4	-0,101	0,064	-0,050	0,050	0,038	0,116	0,190
CTQ-5	-0,214	0,181	0,142	-0,076	0,091	0,247	0,172
CTQ-Total	-0,121	0,138	-0,042	-0,001	0,020	0,169	0,206
PNI-1	0,061	0,059	-0,156	0,151	0,026	0,277	0,132
PNI-2	0,042	-0,009	-0,091	0,184	-0,165	0,160	0,009
PNI-3	0,040	0,048	-0,008	0,033	-0,030	0,167	-0,037
PNI-4	-0,045	-0,103	-0,059	0,039	-0,056	0,204	-0,062
PNI-5	0,058	-0,027	-0,163	0,171	-0,149	0,295	0,061
PNI-6	-0,086	-0,046	-0,099	0,066	-0,046	0,126	-0,066
PNI-7	0,033	-0,045	-0,079	0,146	-0,098	0,275	-0,010
PNI-8	0,064	-0,011	-0,109	0,161	-0,134	0,278	0,042
PNI-9	-0,006	-0,044	-0,126	0,128	-0,054	0,274	0,013
PNI-10	0,018	-0,020	-0,129	0,137	-0,073	0,297	0,016
ESS-1	-0,116	0,023	-0,267	0,122	-0,023	0,217	0,238
ESS-2	-0,064	0,048	-0,237	0,121	0,066	0,245	0,173
ESS-3	-0,159	0,093	-0,159	0,153	0,100	0,219	0,181
ESS-Total	-0,123	0,054	-0,258	0,144	0,038	0,258	0,225
AQ-PA	-0,058	0,157	-0,153	0,050	-0,022	0,235	0,132
AQ-VA	0,007	0,072	-0,003	-0,067	-0,056	0,325	0,054
AQ-A	-0,100	-0,007	-0,096	-0,042	-0,024	0,320	0,194
AQ-H	-0,004	-0,042	-0,153	-0,061	0,053	0,262	0,212
AQ-Total	-0,037	0,032	-0,119	-0,036	-0,005	0,349	0,192
SHI-Total	-0,080	0,141	-0,221	0,190	0,071	0,439	0,190
BPQ-1	-0,186	0,071	-0,192	0,107	-0,018	0,389	0,170
BPQ-2	-0,263	0,076	-0,074	0,013	0,039	0,246	0,143
BPQ-3	-0,075	0,103	-0,176	0,065	0,043	0,269	0,145
BPQ-4	-0,109	0,047	-0,080	-0,022	0,014	0,221	0,140
BPQ-5	-0,117	0,101	-0,228	0,181	0,001	0,113	0,195
BPQ-6	-0,086	0,203	-0,276	0,122	0,101	0,420	0,178
BPQ-7	-0,184	0,121	-0,246	0,136	0,035	0,287	0,170
BPQ-8	-0,172	0,087	-0,143	0,042	-0,003	0,355	0,174
BPQ-9	-0,096	0,042	-0,104	0,071	-0,066	0,237	-0,049
BPQ-Total	-0,187	0,124	-0,226	0,085	0,040	0,379	0,182
BAI-Total	-0,189	0,049	0,073	-0,139	0,108	0,207	0,230
BDI2-Total	-0,188	0,080	-0,182	-0,035	0,132	0,378	0,298

Output of the Web of Science Analysis

Web of Science core collection (all years, search conducted on July 6, 2023)

AGGRESSION AND (INVENTORY OR QUESTIONNAIRE) 9,551

USA3,049
ENGLAND913
SPAIN702
CANADA667
PEOPLES R CHINA566
NETHERLANDS524
GERMANY518
AUSTRALIA459
ITALY421
TURKEY308
SWEDEN226
IRAN195
FRANCE185
POLAND175
JAPAN162
SWITZERLAND161
NORWAY160
ISRAEL156
SOUTH KOREA145
BRAZIL141
RUSSIA141
BELGIUM133
FINLAND130
PORTUGAL120
AUSTRIA98
GREECE94
SCOTLAND93
TAIWAN81
WALES80
INDIA77
MEXICO74
DENMARK69
SOUTH AFRICA64
CROATIA63
CZECH REPUBLIC61
HUNGARY61
NEW ZEALAND59
ROMANIA59
CHILE58
IRELAND58
COLOMBIA53

PAKISTAN47
MALAYSIA40
ARGENTINA37
CYPRUS36
SERBIA36
SLOVAKIA35
SINGAPORE33
UKRAINE32
NIGERIA31
SAUDI ARABIA31
NORTH IRELAND29
LITHUANIA27
SLOVENIA27
EGYPT21
INDONESIA21
THAILAND20
JORDAN18
ESTONIA16
BOSNIA HERCEG15
LEBANON14
PERU13
ECUADOR12
BULGARIA10
QATAR10
VIETNAM10
ICELAND9
KUWAIT9
SRI LANKA9
U ARAB EMIRATES9
BANGLADESH7
KAZAKHSTAN7
LATVIA7
VENEZUELA7
TANZANIA6
TUNISIA6
UGANDA6
BELARUS5
ETHIOPIA5
GHANA5
KOSOVO5
LUXEMBOURG5
JAMAICA4
KENYA4
MOROCCO4
OMAN4
TURKIYE4
HONG KONG3

MOZAMBIQUE3
NAMIBIA3
PHILIPPINES3
ALBANIA2
ARMENIA2
BOTSWANA2
DEM REP CONGO2
LESOTHO2
PALESTINE2
PARAGUAY2
TRINIDAD TOBAGO2
URUGUAY2
AFGHANISTAN1
ARUBA1
AZERBAIJAN1
BAHRAIN1
BENIN1
BOLIVIA1
BURKINA FASO1
BURUNDI1
CAMBODIA1
CAMEROON1
CAPE VERDE1
CAYMAN ISLANDS1
COTE IVOIRE1
CURACAO1
DOMINICAN REP1
FED REP GER1
GEORGIA1
GUYANA1
IRAQ1
KYRGYZSTAN1
LIBYA1
MACEDONIA1
MALTA1
MAURITIUS1
MONTENEGRO1
SAMOA1
SENEGAL1
SURINAME1
UZBEKISTAN1
ZAMBIA1

ANXIETY AND (INVENTORY OR QUESTIONNAIRE) 93,983

USA22,125
ENGLAND9,633
PEOPLES R CHINA6,792
AUSTRALIA6,091
GERMANY6,052
CANADA5,474
NETHERLANDS4,986
TURKEY4,497
SPAIN4,458
ITALY4,131
IRAN3,203
SWEDEN2,820
FRANCE2,686
BRAZIL2,399
JAPAN2,331
SOUTH KOREA1,942
NORWAY1,858
SWITZERLAND1,618
INDIA1,509
ISRAEL1,427
POLAND1,423
BELGIUM1,271
DENMARK1,247
TAIWAN1,165
SCOTLAND1,090
FINLAND989
SAUDI ARABIA964
PORTUGAL946
GREECE849
AUSTRIA785
IRELAND752
MALAYSIA735
WALES675
NEW ZEALAND642
SOUTH AFRICA639
PAKISTAN608
RUSSIA580
SINGAPORE579
MEXICO574
HUNGARY447
ROMANIA428
EGYPT425
INDONESIA358
CHILE332
CZECH REPUBLIC332

CROATIA330
SERBIA324
THAILAND321
NIGERIA311
ARGENTINA263
COLOMBIA257
NORTH IRELAND257
JORDAN256
ETHIOPIA248
SLOVAKIA222
TURKIYE211
U ARAB EMIRATES209
BANGLADESH195
LEBANON186
CYPRUS174
ICELAND158
UKRAINE157
SLOVENIA155
LITHUANIA152
PERU151
VIETNAM134
TUNISIA117
ECUADOR107
QATAR107
PHILIPPINES103
NEPAL90
KUWAIT88
BOSNIA HERCEG85
ESTONIA83
GHANA77
IRAQ76
UGANDA75
MOROCCO72
BULGARIA65
KENYA61
LUXEMBOURG60
OMAN50
LATVIA48
SRI LANKA44
TANZANIA40
BAHRAIN37
PALESTINE35
MALTA31
PARAGUAY31
HONG KONG30
GEORGIA29
SUDAN28

JAMAICA26
CUBA25
KAZAKHSTAN25
CAMEROON24
SYRIA24
VENEZUELA24
BOTSWANA23
MALAWI23
YEMEN23
ZIMBABWE23
BELARUS22
DOMINICAN REP21
AFGHANISTAN19
URUGUAY19
COSTA RICA18
KOSOVO17
ALGERIA16
CAMBODIA16
DEM REP CONGO16
RWANDA16
TRINIDAD TOBAGO16
LIBYA14
MACEDONIA13
NORTH MACEDONIA13
ALBANIA12
BARBADOS12
HONDURAS12
MONTENEGRO12
ARMENIA11
BRUNEI10
ZAMBIA10
AZERBAIJAN9
BENIN9
GIBRALTAR8
GUATEMALA8
MONACO8
BURKINA FASO7
MOZAMBIQUE7
COTE IVOIRE6
FIJI6
MAURITIUS6
MONGOLIA6
MYANMAR6
PANAMA6
SIERRA LEONE6
UZBEKISTAN6
GAMBIA5

GRENADA5
NAMIBIA5
BELIZE4
EL SALVADOR4
KYRGYZSTAN4
YUGOSLAVIA4
ANDORRA3
ANGOLA3
BOLIVIA3
ESWATINI3
FED REP GER3
GABON3
GUYANA3
MOLDOVA3
SENEGAL3
ST KITTS NEVI3
USSR3
ANGUILLA2
ANTIGUA BARBU2
BURUNDI2
CURACAO2
CZECHOSLOVAKIA2
GUINEA2
HAITI2
LAOS2
LIBERIA2
MALDIVES2
NETH ANTILLES2
NEW CALEDONIA2
SURINAME2
ARUBA1
BHUTAN1
CENT AFR REPUBL1
CHAD1
DOMINICA1
ERITREA1
GUINEA BISSAU1
IRAN ISLAMIC R1
LESOTHO1
MALI1
MARSHALL ISLAND1
MICRONESIA1
NICARAGUA1
NIGER1
PAPUA N GUINEA1
REP CONGO1
SINT MAARTEN1

SOMALIA1
SOUTH SUDAN1
ST MARTIN1
TIMOR LESTE1
TOGO1

DEPRESSION AND (INVENTORY OR QUESTIONNAIRE) 121,136

USA35,019
ENGLAND11,682
PEOPLES R CHINA8,358
GERMANY8,195
AUSTRALIA7,534
CANADA7,150
NETHERLANDS5,875
ITALY4,881
TURKEY4,816
SPAIN4,686
JAPAN3,539
SWEDEN3,471
IRAN3,334
BRAZIL3,274
FRANCE3,212
SOUTH KOREA3,193
NORWAY2,344
SWITZERLAND2,262
POLAND1,877
FINLAND1,866
DENMARK1,810
INDIA1,807
TAIWAN1,704
BELGIUM1,637
ISRAEL1,593
SCOTLAND1,298
AUSTRIA1,086
PORTUGAL1,070
SAUDI ARABIA984
GREECE982
SOUTH AFRICA947
IRELAND892
SINGAPORE833
MALAYSIA822
NEW ZEALAND817
WALES764
MEXICO746
PAKISTAN740
HUNGARY577

EGYPT545
RUSSIA526
THAILAND509
NIGERIA487
ETHIOPIA453
CHILE443
CZECH REPUBLIC426
ROMANIA412
SERBIA406
CROATIA387
ARGENTINA327
COLOMBIA327
NORTH IRELAND326
INDONESIA316
JORDAN285
BANGLADESH245
LEBANON240
U ARAB EMIRATES218
PERU214
VIETNAM198
SLOVENIA194
CYPRUS189
UGANDA188
LITHUANIA186
SLOVAKIA186
TURKIYE185
ICELAND183
ESTONIA155
NEPAL147
KENYA142
TUNISIA131
QATAR125
GHANA117
UKRAINE117
PHILIPPINES113
ECUADOR112
KUWAIT100
BOSNIA HERCEG97
BULGARIA97
SRI LANKA93
MOROCCO84
TANZANIA82
IRAQ78
LUXEMBOURG76
MALAWI74
LATVIA54
JAMAICA53

CAMEROON47
PALESTINE46
VENEZUELA44
ZIMBABWE43
OMAN42
SUDAN39
BAHRAIN38
HONG KONG38
RWANDA36
BOTSWANA35
GEORGIA35
TRINIDAD TOBAGO35
CUBA34
KAZAKHSTAN29
URUGUAY29
MALTA28
PARAGUAY27
SYRIA27
AFGHANISTAN25
KOSOVO25
ALBANIA24
DEM REP CONGO22
DOMINICAN REP21
BARBADOS20
ZAMBIA19
BELARUS18
CAMBODIA17
COSTA RICA17
MACEDONIA16
BRUNEI14
COTE IVOIRE13
SENEGAL13
ALGERIA12
BENIN12
NORTH MACEDONIA12
YEMEN12
MOZAMBIQUE11
MYANMAR11
HAITI10
MONTENEGRO10
BOLIVIA9
BURKINA FASO9
GUATEMALA9
HONDURAS9
MONACO9
ARMENIA8
GIBRALTAR8

MOLDOVA8
FIJI7
GAMBIA7
LIBYA7
MAURITIUS7
BURUNDI6
LAOS6
MONGOLIA6
NAMIBIA6
TOGO6
YUGOSLAVIA6
AZERBAIJAN5
GRENADA5
GUINEA5
KYRGYZSTAN5
REP CONGO5
BELIZE4
BHUTAN4
CENT AFR REPUBL4
EL SALVADOR4
ESWATINI4
LESOTHO4
MALI4
PANAMA4
PAPUA N GUINEA4
SIERRA LEONE4
ST KITTS NEVI4
SURINAME4
UZBEKISTAN4
ANDORRA3
ANGOLA3
ANTIGUA BARBU3
BAHAMAS3
ERITREA3
LIBERIA3
MADAGASCAR3
MALDIVES3
NIGER3
SERBIA MONTENEG3
SOMALIA3
ANGUILLA2
CURACAO2
GABON2
GUYANA2
NETH ANTILLES2
NICARAGUA2
SOLOMON ISLANDS2

BERMUDA1
DJIBOUTI1
FED REP GER1
FRENCH GUIANA1
GUINEA BISSAU1
IRAN ISLAMIC R1
MICRONESIA1
NEW CALEDONIA1
SAN MARINO1
SEYCHELLES1
SINT MAARTEN1
ST MARTIN1
ST VINCENT1
SWAZILAND1
TIMOR LESTE1
USSR1

BORDERLINE PERSONALITY AND (INVENTORY OR QUESTIONNAIRE) 3,185

USA1,128
GERMANY437
ENGLAND274
CANADA237
ITALY209
NETHERLANDS187
AUSTRALIA173
SPAIN161
SWITZERLAND128
FRANCE111
TURKEY89
BELGIUM87
PEOPLES R CHINA85
NORWAY70
IRAN65
AUSTRIA60
SWEDEN59
DENMARK56
POLAND39
HUNGARY34
ISRAEL31
SCOTLAND31
BRAZIL30
JAPAN30
FINLAND27
NEW ZEALAND25
IRELAND20
MEXICO18

SOUTH KOREA17
TAIWAN17
CZECH REPUBLIC15
GREECE15
PORTUGAL15
WALES15
SOUTH AFRICA13
EGYPT11
INDIA11
RUSSIA10
CROATIA8
NORTH IRELAND8
ARGENTINA7
LUXEMBOURG7
THAILAND7
PAKISTAN6
CHILE5
SERBIA5
SINGAPORE5
SLOVENIA4
URUGUAY4
BOSNIA HERCEG3
LITHUANIA3
MOROCCO3
SAUDI ARABIA3
U ARAB EMIRATES3
COLOMBIA2
ETHIOPIA2
SLOVAKIA2
UKRAINE2
YUGOSLAVIA2
ANDORRA1
BARBADOS1
ECUADOR1
GIBRALTAR1
ICELAND1
IRAQ1
JORDAN1
LATVIA1
LEBANON1
MALAYSIA1
MAURITIUS1
MOLDOVA1
PANAMA1
PERU1
ROMANIA1
SUDAN1

TUNISIA1
TURKIYE1

NARCISSISM AND (INVENTORY OR QUESTIONNAIRE) 1,939

USA828
GERMANY187
ENGLAND171
CANADA158
AUSTRALIA99
PEOPLES R CHINA95
ITALY83
POLAND81
NETHERLANDS70
ISRAEL56
SPAIN53
TURKEY43
AUSTRIA36
IRAN35
SWITZERLAND30
JAPAN24
RUSSIA24
SWEDEN22
CROATIA20
NORWAY19
SERBIA19
BELGIUM18
DENMARK18
SOUTH KOREA18
MALAYSIA16
PORTUGAL16
SCOTLAND16
BRAZIL13
CZECH REPUBLIC13
NEW ZEALAND13
SINGAPORE13
WALES13
FRANCE12
HUNGARY12
FINLAND11
PAKISTAN11
GREECE10
INDIA10
NORTH IRELAND10
ROMANIA10
ARGENTINA9
TAIWAN7

INDONESIA6
LITHUANIA5
MEXICO5
SLOVAKIA5
U ARAB EMIRATES5
UKRAINE5
ESTONIA4
JORDAN4
SAUDI ARABIA4
SLOVENIA4
THAILAND4
CHILE3
EGYPT3
IRELAND3
LEBANON3
LUXEMBOURG3
SOUTH AFRICA3
BOSNIA HERCEG2
CYPRUS2
GEORGIA2
KUWAIT2
MOROCCO2
PERU2
QATAR2
TOGO2
TUNISIA2
VIETNAM2
AZERBAIJAN1
BELARUS1
BRUNEI1
BULGARIA1
COLOMBIA1
GIBRALTAR1
ICELAND1
KYRGYZSTAN1
LIECHTENSTEIN1
NIGERIA1
PALESTINE1
PHILIPPINES1
SENEGAL1
ZIMBABWE1

SELF-HARM AND (INVENTORY OR QUESTIONNAIRE) 1815

USA496
ENGLAND375
AUSTRALIA200

PEOPLES R CHINA118
CANADA116
GERMANY99
ITALY77
SWEDEN69
NETHERLANDS66
IRELAND62
NORWAY59
SPAIN59
BELGIUM48
TURKEY47
SCOTLAND44
FRANCE43
JAPAN42
FINLAND34
NEW ZEALAND30
IRAN29
ISRAEL29
SWITZERLAND29
TAIWAN29
DENMARK28
INDIA28
HUNGARY27
SOUTH KOREA26
AUSTRIA24
PORTUGAL20
WALES20
POLAND19
RUSSIA19
BRAZIL17
PAKISTAN16
MALAYSIA14
MEXICO14
ROMANIA14
SLOVENIA13
SOUTH AFRICA11
NIGERIA10
NORTH IRELAND10
ESTONIA9
GREECE9
SINGAPORE9
SRI LANKA9
CHILE8
CZECH REPUBLIC7
ICELAND6
LITHUANIA6
BULGARIA5

CROATIA5
ETHIOPIA5
INDONESIA5
JORDAN5
SLOVAKIA5
TUNISIA5
BANGLADESH4
COLOMBIA4
CYPRUS4
MOROCCO4
THAILAND4
EGYPT3
GHANA3
KAZAKHSTAN3
LEBANON3
PERU3
SAUDI ARABIA3
U ARAB EMIRATES3
UGANDA3
ARGENTINA2
ECUADOR2
LATVIA2
TANZANIA2
TURKIYE2
UKRAINE2
VIETNAM2
AFGHANISTAN1
AZERBAIJAN1
BELARUS1
BOSNIA HERCEG1
BOTSWANA1
CAMBODIA1
GEORGIA1
GUINEA1
HONDURAS1
IRAQ1
KYRGYZSTAN1
LIBERIA1
LIBYA1
LUXEMBOURG1
MALI1
NEPAL1
NICARAGUA1
PARAGUAY1
PHILIPPINES1
QATAR1
RWANDA1

SERBIA1
SIERRA LEONE1
ZIMBABWE1

SHAME AND (INVENTORY OR QUESTIONNAIRE) 1658

USA484
ENGLAND172
CANADA126
GERMANY126
AUSTRALIA117
NETHERLANDS89
PEOPLES R CHINA70
ITALY66
PORTUGAL62
SPAIN49
SWEDEN48
ISRAEL47
IRAN45
NORWAY44
TURKEY40
FRANCE39
BRAZIL36
POLAND36
SWITZERLAND34
BELGIUM31
JAPAN26
SCOTLAND21
SOUTH KOREA20
AUSTRIA18
DENMARK18
INDIA15
FINLAND14
CROATIA13
MEXICO13
HUNGARY12
NEW ZEALAND12
SOUTH AFRICA12
TAIWAN11
NIGERIA10
RUSSIA10
SINGAPORE10
PAKISTAN9
SAUDI ARABIA9
ROMANIA8
JORDAN7
NORTH IRELAND7

WALES7
GREECE6
INDONESIA6
IRELAND6
MALAYSIA6
THAILAND6
CZECH REPUBLIC5
PHILIPPINES5
BANGLADESH4
CHILE4
LUXEMBOURG4
SLOVENIA4
ARGENTINA3
COLOMBIA3
EGYPT3
ETHIOPIA3
LEBANON3
MOROCCO3
SERBIA3
SLOVAKIA3
U ARAB EMIRATES3
UGANDA3
BOSNIA HERCEG2
ICELAND2
KENYA2
LATVIA2
LITHUANIA2
NEPAL2
PERU2
SRI LANKA2
TANZANIA2
TUNISIA2
BAHAMAS1
BELARUS1
COSTA RICA1
CYPRUS1
GEORGIA1
GHANA1
GRENADA1
GUATEMALA1
IRAQ1
JAMAICA1
KAZAKHSTAN1
NORTH MACEDONIA1
OMAN1
PAPUA N GUINEA1
RWANDA1

SURINAME1
UKRAINE1
URUGUAY1
VIETNAM1
ZAMBIA1
ZIMBABWE1

CHILDHOOD TRAUMA AND (INVENTORY OR QUESTIONNAIRE) 5118

USA1,722
GERMANY613
PEOPLES R CHINA457
ENGLAND422
CANADA386
NETHERLANDS327
TURKEY320
AUSTRALIA245
ITALY193
BRAZIL179
SWITZERLAND164
FRANCE130
SOUTH KOREA125
SPAIN125
NORWAY121
SWEDEN114
ISRAEL104
SOUTH AFRICA84
AUSTRIA83
SCOTLAND70
BELGIUM69
POLAND64
DENMARK63
JAPAN62
IRAN60
IRELAND59
NORTH IRELAND40
FINLAND39
PORTUGAL34
WALES31
INDIA29
CZECH REPUBLIC26
HUNGARY26
SINGAPORE24
CHILE23
SLOVAKIA21
NEW ZEALAND19
MEXICO18

TAIWAN16
CROATIA15
SERBIA13
GREECE12
TURKIYE12
SAUDI ARABIA11
THAILAND11
EGYPT10
MALAYSIA10
NIGERIA10
ARGENTINA9
BOSNIA HERCEG9
ICELAND9
KENYA9
ESTONIA8
JORDAN8
LEBANON8
RUSSIA8
TANZANIA8
UGANDA8
COLOMBIA7
CYPRUS7
MONACO7
ROMANIA7
SLOVENIA7
INDONESIA6
TUNISIA6
LITHUANIA5
PAKISTAN5
PERU5
TRINIDAD TOBAGO5
BANGLADESH4
IRAQ4
KAZAKHSTAN4
NEPAL4
U ARAB EMIRATES4
VIETNAM4
BARBADOS3
BULGARIA3
CAMBODIA3
ETHIOPIA3
LUXEMBOURG3
SRI LANKA3
URUGUAY3
CAMEROON2
ECUADOR2
GEORGIA2

KOSOVO2
MOROCCO2
PALESTINE2
TOGO2
UKRAINE2
ZIMBABWE2
ALBANIA1
BELARUS1
BURKINA FASO1
BURUNDI1
DEM REP CONGO1
EL SALVADOR1
GHANA1
JAMAICA1
LATVIA1
MACEDONIA1
MALAWI1
MALTA1
MONTENEGRO1
MYANMAR1
PARAGUAY1
SOMALIA1
SUDAN1
SYRIA1
TIMOR LESTE1
